# Exploration of the causes of cerebrospinal fluid leakage after endoscopic endonasal surgery for sellar and suprasellar lesions and analysis of risk factors

**DOI:** 10.3389/fsurg.2022.981669

**Published:** 2022-09-13

**Authors:** Yicheng Xiong, Yajing Liu, Guo Xin, Shenhao Xie, Hai Luo, Liming Xiao, Xiao Wu, Tao Hong, Bin Tang

**Affiliations:** ^1^ Department of Neurosurgery, The First Affiliated Hospital of Nanchang University, Nanchang, China; ^2^ Operating Theater, The First Affiliated Hospital of Nanchang University, Nanchang, China

**Keywords:** endoscopic endonasal surgery, risk factors, cerebrospinal fluid leakage, postoperative leakage, skull base reconstruction, bony reconstruction

## Abstract

**Objective:**

Postoperative cerebrospinal fluid (CSF) leakage following endoscopic endonasal surgery (EES) is a frequent complication. This study aims to identify potential risk factors of postoperative CSF leakage.

**Methods:**

A retrospective review of 360 patients who underwent EES was included. The associations between postoperative CSF leakage and patient demographics, medical history, tumor characteristics, and intraoperative repair techniques were analyzed; the diagnosis and repair of postoperative CSF leakage were also introduced.

**Results:**

Postoperative CSF leakage occurred in 14 patients (3.9%), 2 of them cured by lumbar cistern drainage, 12 underwent endoscopic repair. Among these 12 cases, 3 were repaired twice, and the rest were cured the first time. During the repair surgery, insufficient embedded fat was detected in one case detected, seven with breached inner artificial dura, three had vascularized pedicle nasoseptal flap (VP-NSF) displacement, two with VP-NSF perforation, two with VP-NSF inactivation, and one with imperfect adherence to VP-NSF to the skull base. Eight cases had intracranial infections. Excluding one case who died of severe intracranial infection, the rest were cured and discharged without obvious sequelae. Multivariate analysis revealed that the suprasellar lesion, subarachnoid invasion, and intraoperative grade 3 flow CSF leakage were the risk factors of CSF leakage after operation, while the bone flap was a protective factor.

**Conclusion:**

Bone flap combined with VP-NSF and iodoform gauze for skull base reconstruction is recommended in high-risk patients, while postoperative lumbar cistern drain remains dispensable.

## Introduction

Cerebrospinal fluid (CSF) leakage is a pathologic condition where CSF flows out from defects of the dural and skull base, and it can be caused by a multitude of different factors, mainly including trauma (1) and endoscopic endonasal surgery (EES). Although with the recent development of neuroendoscopic equipment, intraoperative hemostatic materials, and the concept of skull base reconstruction, EES has been employed in various types of skull base tumors. EES (including expend EES) allows tumor removal at anterior skull base, parasellar, suprasellar, and petroclival regions from the midline access. Noteworthy, as one of the most common complications in EES (2–4), CSF leakage after operation not only increases the duration of hospital stay (5) and readmission rates but also increases the risk of postoperative intracranial infection and seriously affects the prognosis (6). It has been reported that postoperative CSF leakage ranges from 7.2% to 25.4% (7–11), making EES questionable.

While several studies have reported the factors affecting postoperative CSF leakage, discussion related to the reconstruction defects is really rare. Here, we investigated the risk factors of postoperative CSF leakage through systematic analysis and discussed the defects in reconstruction technique. We hope our research could serve as a reference for the progression of EES.

## Materials and methods

### Data collection

A total of 360 patients who underwent EES were selected from the Department of Neurosurgery, the First Affiliated Hospital of Nanchang University, including 184 males and 176 females. Ages ranged from 4–81 years, with an average of (46 ± 14) years, including craniopharyngiomas (*n* = 57), pituitary adenomas (*n* = 264), tuberculum sellae meningioma (*n* = 32), and Rathke’s cysts (*n* = 7).

### Surgical procedure

**Figure 1:**
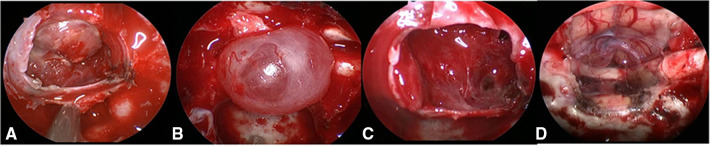
Grading of CSF leakage during operation. (**A**) Grade 0: the sellar diaphragm was intact and no CSF leakage after tumor resection. Postoperative pathology showed that one case had no functional pituitary adenoma. (**B**) Grade 1: the sellar diaphragm was intact and a small vesicle with CSF accumulation was formed around it after tumor resection. Postoperative pathology showed one case of nonfunctional pituitary adenoma. (**C**) Grade 2: the sellar diaphragm defect and moderate CSF leakage can be observed after tumor resection. Pathology showed one case of nonfunctional pituitary adenoma. (**D**) Grade 3; postoperative pathology showed one case craniopharyngioma of with extensive suprasellar arachnoid cistern opening during operation. CSF, cerebrospinal fluid.

Two-person/three-hand or the two-person/four-hand technique was used in EES. Decision of harvesting a vascularized pedicle nasoseptal flap (VP-NSF) or a free mucosal flap was made according to the surgery approach (classic EES or expend EES). After harvesting the NSF, the posterior nasal tract was opened and the nasal septum bone flap was made; the details are described in our previous article (12). Wide opening of the sphenoid sinus after the nasal procedure, exposing the posterior and lateral walls of the sphenoid sinus, with the sellar floor at the center, the sphenoethmoid planum above, and the clival indentation below. For creating a bone flap *in situ* (ISBF), the details are described in the article by Jin et al. (13) with intraoperative CSF leakage after lesion removal. The classification of intraoperative flow CSF leakage was defined as follows: grade 0:absence of CSF leakage, with intact sellar diaphragma; grade 1: small “weeping” leak, with only tiny diaphragmatic defect; grade 2: obvious defect of sellar diaphragma or skull base dura mater with moderate CSF exudation; grade 3:high-flow CSF leak, large sellar diaphragmatic or skull base dura defect with the total opening of the suprasellar arachnoid cistern and/or opening of the floor of the third ventricle (14). See Figure 1 for details. Meanwhile, we performed multilayer skull base reconstruction according to intraoperative flow of CSF leakage. As for the reconstruction steps of intraoperative grade 3 flow CSF leakage, we changed before and after September 2018 as shown in Table 1. The details of bone flap placement are as follows: (1) After artificial dural embedding, the nasal septum bone flap was trimmed based on the shape and size of the skull base bone window to repair the bone defect, placement not inside or outside but just at the same plane with the skull base for the optimized simulation of the inherent anatomical structure. (2) After artificial dural embedding, the ISBF was gently countersunk into the bone defect; then, several points of the edge of the ISBF were wedged between the dura and bone for fixation (13).

**Table 1:** Cerebrospinal fluid leak repair protocol.

January 2018–August 2018	
Grade of leakage	Repair method
0	Collagen sponge + free mucosal graft + iodoform gauze support
1	Collagen sponge + artificial dura + VP-NSF + iodoform gauze support
2	Autologous fat graft + artificial dura + VP-NSF + balloon support
3	Autologous fat graft + artificial dura mater + fascia lata + VP-NSF + balloon support + lumbar cistern drainage for 72 h (Figure 2)
September 2018–December 2020	
Grade of leakage	Repair method
0	The same as above
1	Collagen sponge + artificial dura + *in situ* bone flap or nasal septum bone flap + VP-NSF + iodoform gauze support
2	Autologous fat graft + artificial dura + *in situ* bone flap or nasal septum bone flap + VP-NSF + iodoform gauze support
3	Autologous fat graft + artificial dura + *in situ* bone flap or nasal septum bone flap + VP-NSF + iodoform gauze support (Figures 3, 4)

VP-NSF,  vascularized pedicle nasoseptal flap.

**Figure 2:**
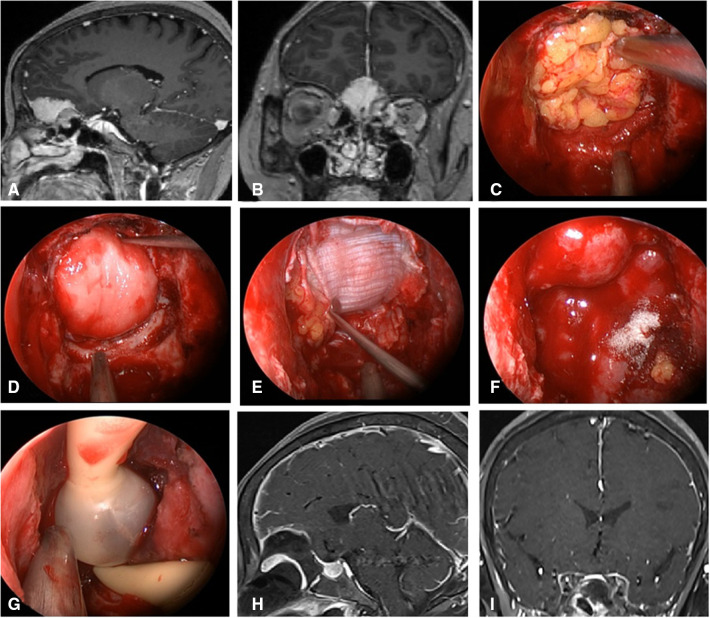
Changes in reconstruction strategy/(before). (**A,B**) Sagittal and coronal enhanced MRI in sellar region before operation, and olfactory groove meningioma was considered. (**C**) Subdural fat packing. (**D**) Artificial dura mater embedded between cellulite and dura mater. (**E**) Cover the fascia lata on the artificial dura mater. (**F**) Cover the VP-NSF on the fascia lata. (**G**) Balloon support. (**H,I**) Sagittal and coronal enhanced MRI in sellar region after operation, and postoperative pathology showed meningioma.

**Figure 3:**
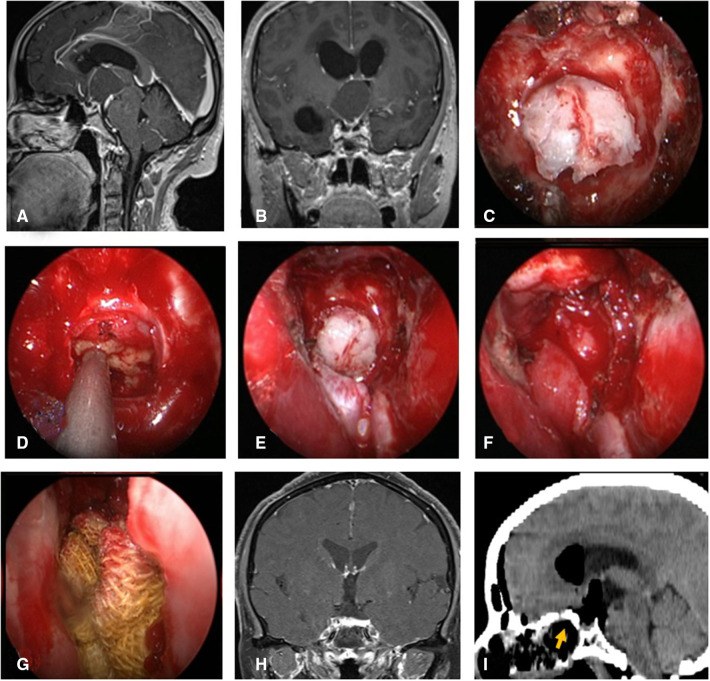
Changes in reconstruction strategy/(after) [(**C**) ISBF harvesting; (**D–G**) process of reconstruction]. (**A,B**) MRI enhanced in sellar region before operation, and craniopharyngioma was considered; .**C**) Osteoclastic craniectomy to creating a bone flap *in situ*. (**D**) Subdural fat packing. (**E**) Repair of skull base bone window with ISBF. (**F**) Cover the VP-NSF on the ISBF. (**G**) Gauze support. (**H**) Coronal enhanced MRI in sellar after operation and postoperative pathology showed craniopharyngioma. (**I**) CT bone window showing ISBF was in place. VP-NSF, vascularized pedicle nasoseptal flap, ISBF, *in situ* bone flap.

**Figure 4:**
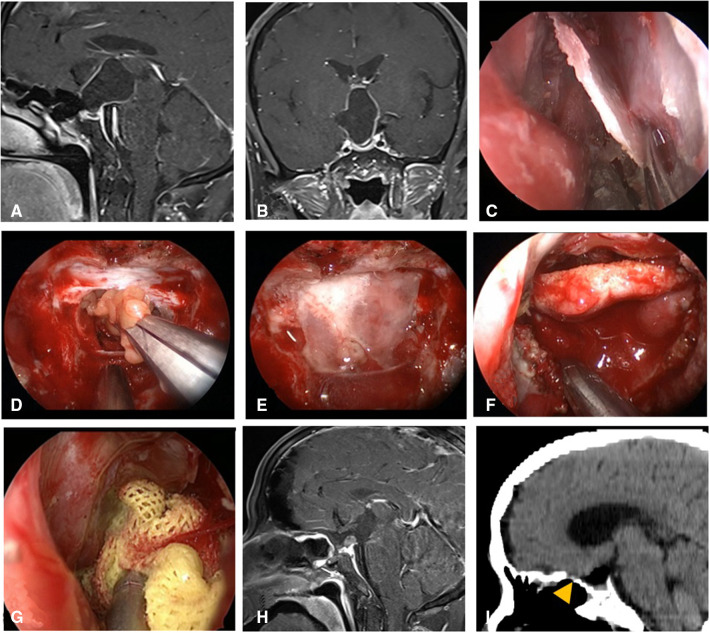
Changes in reconstruction strategy/(after) [(**C**) harvest of nasal septum bone flap; (**D–G)** process of reconstruction]. (**A,B**) MRI enhanced in sellar region before operation, and craniopharyngioma was considered before operation. (**C**) Separation of nasal septum bone flap. (**D**) Subdural fat packing. (**E**) Repair of skull base bone window with trimmed nasal septum bone flap. (**F**) Cover the VP-NSF on the nasal septum bone flap. (**G**) Gauze support. (**H**) MRI enhanced in sellar after operation and postoperative pathology showed craniopharyngioma. (**I**) CT bone window showing nasal septum bone flap was in place. VP-NSF, vascularized pedicle nasoseptal flap.

### Diagnosis and management of postoperative CSF leakage

For all patients, CT scan was typically performed within 6 h postoperatively. MRI of the sellar region was reexamined within 3 days. The nasal packing was removed at about 5–7 days postoperatively for patients with grade 0 and 1 CSF leakage during operation and at 12–14 days postoperatively for those with grade 2 and 3. Endoscopic nasal cleaning was performed 2, 4, and 6 weeks after nasal packing removal. CT examination was conducted first to determine the presence of neurocranium if patients were suspected of postoperative CSF leakage and then endoscopic re-exploration was done as soon as possible. The diagnosis of postoperative CSF leakage is as follows: (1) Patients with clear liquid flow out from the nasal cavity after operation. CSF routine test, biochemical parameters (15), *β*-2 transferrin (16), and *β*-trace protein (17) examination of the liquid sample should be performed immediately, and CT scan should be performed to exclude intracranial pneumatosis. (2) Patients with no clear fluid flow from the nasal cavity after operation; patients complaining of itching in the throat, a foreign body sensation, and salty water flowing down the posterior pharynx should be suspected of CSF leakage. In addition, patients with recurrent postoperative fever, uncontrollable pulmonary infection, and clinical features of intracranial infection should also be suspected. Early endoscopic exploration for CSF leakage suspects is absolutely advocated, and prompt endoscopic repair after clear diagnosis is necessary.

### Statistical analysis

All statistical analyses were performed using SPSS version 26 (IBM Corporation, USA). The continuous variables conforming to the normal distribution were expressed by mean ± standard deviation (M ± SD). An independent-sample *t*-test was used for comparison between the two groups. The number of cases or percentages is expressed in the classified data. Chi-square tests were used for comparison between groups, and group comparisons were made with chi-square or Fisher’s exact test in cases with a small number of expected outcomes. All independent variables thought to be of clinical significance *a priori* were placed into a logistic multiple regression model. *P* value <0.05 was considered statistically significant for all statistical tests.

The statistical steps were mainly divided into two steps: first, age, gender, and tumor type were included as influencing factors in the univariate and multifactor analysis, and then the univariate and multifactor analysis were done separately for different types of tumors.

## Results

### Results and causes of postoperative CSF leakage

**Figure 5:**
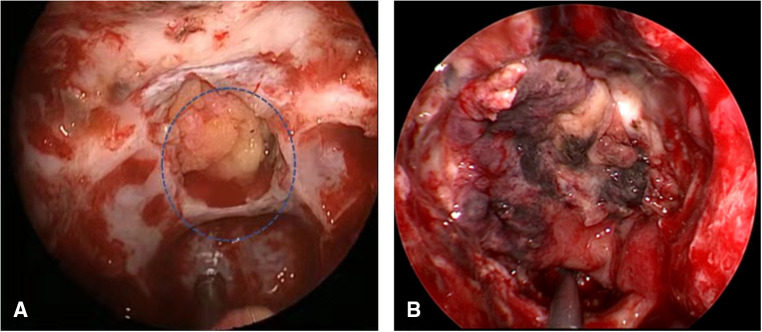
(**A**) Insufficient embedded fat (**circle**). (**B**) VP-NSF inactivation. VP-NSF, vascularized pedicle nasoseptal flap.

Of the cases, 3.9% had postoperative CSF leakage (14/360), including four cases of craniopharyngioma, eight cases of pituitary adenoma, and two cases of sellar tubercle meningioma. Intraoperative CSF leakage of grade 3 was found in 10 cases, grade 2 in 3 cases, and grade 1 in 1 case. Among these, nine cases of postoperative CSF leakage occurred within 14 days, five cases occurred within 15–30 days, and the average time was (13 ± 5) days. Among all, 2 cases were cured by lumbar drainage, and the other 12 cases were explored under endoscopy. We found that the leading cause of postoperative CSF leakage was inadequate reconstruction, including one case with insufficient embedded fat (Figure 5A), seven cases with breached inner artificial dura, three cases with VP-NSF displacement, two cases with VP-NSF inactivation (Figure 5B), two cases with VP-NSF perforation, and one case with imperfect adherence of VP-NSF to the skull base (Table 2).

**Table 2:** Causes of CSF after endoscopic endonasal surgery.

ID	Sex /age	Pathology	ICSF leakage flow grading	Causes	Time	Repair times	Nasal packing	Complication
1	M/29	Pituitary adenoma	Grade 3	Insufficient embedded fat	14	1	B	Intracranial infection
2	F/52	Meningioma	Grade 3	Inner artificial dura breach	15	1	G	—
3	F/50	Pituitary adenoma	Grade 2	VP-NSF perforation	23	1	B	—
4	M/45	Pituitary adenoma	Grade 3	VP-NSF Perforation	20	2	B	—
5	M/19	Craniopharyngioma	Grade 3	Inner artificial dura breach + Not firm adherence of VP-NSF to the skull base	19	2	B	Intracranial infection
6	F/57	Craniopharyngioma	Grade 3	Inner artificial dura breach	12	1	B	Intracranial infection
7	F/48	Pituitary adenoma	Grade 3	VP-NSF displacement + Inner artificial dura breach	5	1	B	—
8	M/53	Pituitary adenoma	Grade 2	Inner artificial dura breach	9	1	B	—
9	M/58	Pituitary adenoma	Grade 3		10	1	G	Intracranial infection
10	F/63	Craniopharyngioma	Grade 3	Inner artificial dura breach + VP-NSF displacement	14	2	B	Intracranial infection
11	F/61	Pituitary adenoma	Grade 3	VP-NSF inactivation + VP-NSF displacement	13	1	G	Intracranial infection
12	M/42	Meningioma	Grade 3	Inner artificial dura breach	15	1	B	Intracranial infection
13	M/22	Craniopharyngioma	Grade 2	LD < 72 h	3	0	G	Intracranial infection
14	F/43	Pituitary adenoma	Grade 1	LD < 72 h	14	0	B	/

Grade 1 = small “weeping” leak, without obvious or with only small diaphragmatic defect; grade 2 = obvious defect of sellar diaphragma or skull base dura mater with moderate CSF exudation; grade 3 = large CSF leak, large sellar diaphragmatic or skull base dural defect with extensive opening of suprasellar arachnoid cistern and/or opening of the floor of the third ventricle.

B, balloon; G, gauze; LD, lumbar cistern drainage; VP-NSF, vascularized pedicle nasoseptal flap; CSF, cerebrospinal fluid; ICSF, intraoperative cerebrospinal fluid.

### Treatment of postoperative CSF leakage

**Figure 6:**
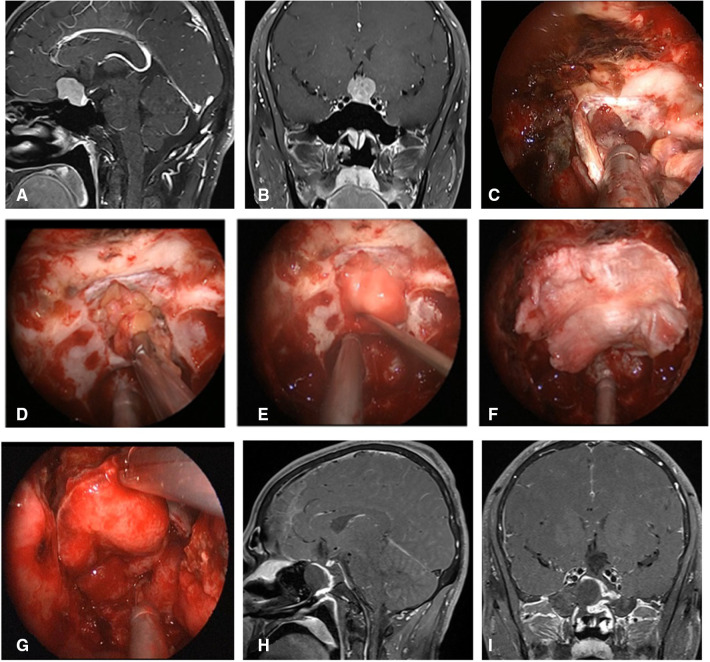
Repair process of patient with insufficient embedded fat. (**A,B**) MRI enhanced in sellar region before operation and was considered pituitary adenoma. (**C**) Uncover the VP-NSF to see the subdural leakage. (**D**) Subdural fat packing. (**E**) Artificial dura mater embedded between cellulite and dura mate. (**F**) Cover fascia lata on the artificial inlay dura (arrow). (**E**) Cover the VP-NSF on the fascia lata. (**H,I**) Sagittal and coronal enhanced MRI in sellar region after operation, and postoperative pathology showed pituitary adenoma. VP-NSF, vascularized pedicle nasoseptal flap.

**Figure 7:**
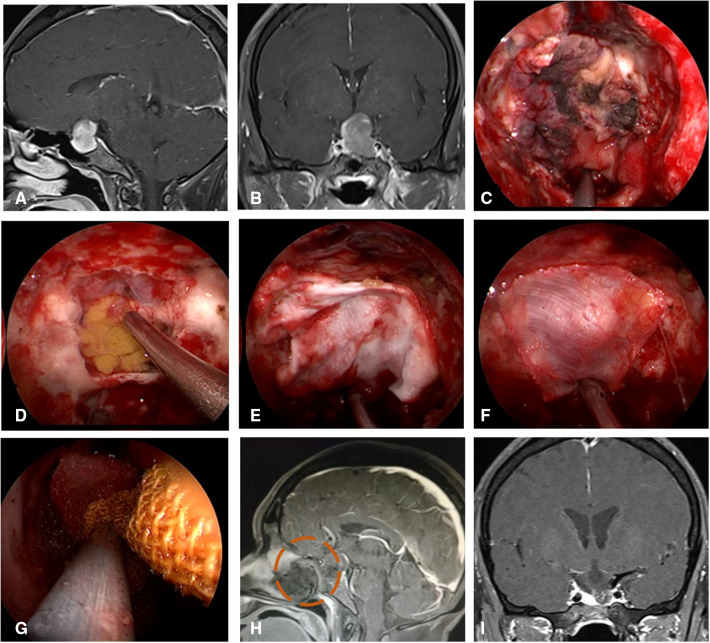
Repair process of patient with VP-NSF inactivation. (**A,B**) MRI enhanced in sellar region before operation and was considered pituitary adenoma. (**C**) VP-NSF inactivation observed on the endoscopy (black area). (**D**) Unraveling the artificial inner dura. (**E**) Artificial dura mater embedded between cellulite and dura mater. (**F**) Replacement of inactivated nasal septal mucosal flap using fascia lata. (**G**) Collagen sponge and biological protein glue was fixed and then supported with iodoform gauze. (**H**) MRI enhancement at postoperative week 2 showed no significant enhancement of VP-NSF (circle). (**I**) Postoperative MRI enhancement in sellar region. VP-NSF, vascularized pedicle nasoseptal flap.

Two of the 14 patients with postoperative CSF leakage were cured by lumbar cistern drainage (LD). Twelve patients underwent endoscopic exploration and repair. The principle of repair was to determine the causes and then repair. When the cause was determined to be insufficient embedded fat, refilling the subdural leak with fat is necessary (Figure 6). When faced with the breached inner artificial dura, the artificial dura was reinserted between cellulite and dura mater. In case of VP-NSF inactivation, fascia lata was used instead (Figure 7). Necessity of LD was based on the flow of CSF leakage during the repair. Three patients were repaired twice to resolve the postoperative CSF leakage, while the others were cured after the first time. Eight cases were complicated with intracranial infection; except for one case who died of severe intracranial infection, the rest were cured and discharged without obvious sequelae.

### Univariate and multivariate analysis of postoperative CSF leakage

The univariate analysis described that craniopharyngioma, pituitary adenoma, lesions in the sellar or suprasellar region, subarachnoid invasion, intraoperative CSF leakage, bony reconstruction, balloon support, and postoperative LD were significantly correlated with postoperative CSF leakage. In addition, age, sex, hypertension, diabetes, radiotherapy, revision surgery, and maximum tumor diameter were not significantly associated with postoperative CSF leakage (Table 3). Further multivariate logistic regression analysis confirmed that the lesion was located on the suprasellar [odds ratio (OR) = 3.690, 95% CI: 1.029–5.783, *P* = 0.003] or subarachnoid space invasion (OR = 4.879, 95% CI: 1.243–12.820, *P* = 0.007); intraoperative grade 3 CSF leakage flow was the risk factor CSF leakage after EES (OR = 7.392, 95% CI: 2.458–19.736, *P* = 0.012), while bony reconstruction (OR = 0.313, 95% CI: 0.099–0.694, *P* = 0.019) was the protective factor. Tumor types, balloon support, postoperative LD, and sellar lesion were not significantly correlated with CSF leakage after the operation (Table 4).

**Table 3:** Univariate analysis of factors affecting occurrence of postoperative CSF leakage.

Patient/tumor characteristics	CSF leakage (*n* = 14)	No CSF leakage (*n* = 346)	*χ*^2^/*t*	*P* value
Sex, M/F	7/7	177/169	0.013	0.908
Age, years	44 ± 15	45 ± 14	0.365	0.809
Hypertension	0 (0)	43 (12)	Fisher	0.688
Diabetes	2 (14.3)	16 (4.6)	Fisher	0.162
Radiotherapy	0 (0)	6 (1.7)	Fisher	1.000
Revision surgery	1 (7.1)	47 (13.6)	Fisher	0.704
Pathology				
Craniopharyngioma	4 (29)	53 (15.3)	Fisher	0.003
Pituitary adenoma	8 (57.1)	256 (74.0)	7.038	0.015
Meningioma	2 (18.8)	30 (8.7)	Fisher	0.634
Rathke’s cleft cyst	0 (0)	7 (2.0)	Fisher	1.000
Location				
Sellar	1 (7.1)	192 (55.5)	11.349	<0.001
Suprasellar	13 (81.2)	154 (44.5)	20.204	<0.001
Subarachnoid space invasion	10 (71.4)	100 (28.9)	12.474	<0.001
Maximal tumor diameter, cm	3.0 ± 0.6	2.9 ± 1.2	0.577	0.068
ICSF leakage	14 (100)	182 (52.6)	9.458	0.002
Grade 1	1 (6.3)	34 (9.8)		
Grade 2	3 (21.4)	94 (27.2)		
Grade 3	10 (71.4)	54 (15.6)		
Foley balloon support	10 (71.4)	73 (21.0)	Fisher	0.002
Bony reconstruction	1 (7.1)	103 (29.7)	Fisher	0.013
Postoperative lumbar drainage	9 (64.3)	41 (11.8)	Fisher	<0.001

Bold indicates significance.

Sellar lesions include lesions in the intrasellar and cavernous sinus.

Grade 1 = small “weeping” leak, without obvious or with only small diaphragmatic defect; grade 2 = obvious defect of sellar diaphragma or skull base dura mater with moderate CSF exudation; grade 3 = large CSF leak, large sellar diaphragmatic or skull base dural defect with extensive opening of suprasellar arachnoid cistern and / or opening of the floor of the third ventricle.

F, female; M, male; CSF, cerebrospinal fluid; ICSF, Intraoperative cerebrospinal fluid.

**Table 4:** Multivariate analysis for postoperative CSF leakage.

Factors	OR	95% CI	*P*
Subarachnoid invasion	4.879	1.243–12.820	0.007
Suprasellar lesion	3.690	1.029–5.783	0.003
Intraoperative flow CSF leakage			
Grade 1	2.387	1.085–4.783	0.128
Grade 2	5.442	1.781–14.021	0.111
Grade 3	7.392	2.458–19.736	0.012
Bony reconstruction	0.313	0.099–0.694	0.019

^a^Bold indicates significance.

CI, confidence interval; CSF, cerebrospinal fluid; OR, odds ratio.

**Grade 1 = small “weeping” leak, without obvious or with only small diaphragmatic defect; grade 2 = obvious defect of sellar diaphragma or skull base dura mater with moderate CSF exudation;* *grade 3 = large* *CSF* *leak, large sellar* *diaphragmatic* *or skull base dural defect with extensive opening of* *suprasellar arachnoid* *cistern and/or opening of the floor of the third ventricle.**

## Discussion

Although the incidence of postoperative CSF leakage was significantly reduced by 5%–10% with reconstruction using the VP-NSF multilayer reconstruction technique (8, 12–14), the complications still remained unacceptable. Thus, it is imperative to explore the causes of postoperative CSF leakage and potential influencing factors.

### Endoscopic exploration of patients with postoperative CSF leakage

In this study, we found that postoperative CSF leakage was more common in patients with intraoperative grade 3 flow CSF leakage (10/14), and the leading causes of postoperative CSF leakage were insufficient subdural and epidural reconstruction in multilayer skull base reconstruction. The details are as follows: (1) Insufficient subdural reconstruction due to the inadequate embedded fat and inlaid artificial dura. Inlaid artificial dura is easily washed away in grade 3 CSF leakage flow from the suprasellar arachnoid cistern and even the third ventricle. (2) Insufficient epidural reconstruction, including displacement, necrosis, perforation of the VP-NSF, and imperfect adherence of VP-NSF to the skull base. Displacement of VP-NSF is always caused by improper support of the balloon and inadvertent removal of nasal packing. VP-NSF necrosis usually resulting from impaired vascular pedicle, including irregular nasoseptal flap (NSF) harvesting, high-pressure nasal packing, and sharp bone protuberances of the sphenoid sinus.

### Predictors of postoperative CSF leakage

#### Suprasellar lesion

Multivariate analysis revealed suprasellar lesion as a risk factor for postoperative CSF leakage. The possible reason is that the suprasellar lesion invades the suprasellar arachnoid cistern or even the floor of the third ventricle; tumor removal might result in opening of the floor of the three ventricles and the suprasellar arachnoid cistern while causing a large skull base defect, which in turn leads to intraoperative grade 3 flow CSF leakage, which leads to the occurrence of postoperative CSF leakage.

#### Subarachnoid space invasion

Skull base tumors sometimes invade the bone, dura mater, subarachnoid space, arachnoid cistern, and even protrude into the third ventricle. During the operation of pituitary adenoma, we observed a barrier composed of dura mater, with or without pituitary gland tissue, or arachnoid between tumors and CSF. Tumor invasion to the arachnoid might weaken the anti-CSF barrier and leads to postoperative CSF leakage. It is reported that Villalonga et al. (18) developed a model for predicting intraoperative and postoperative CSF leakage; the results confirmed a significant correlation between subarachnoid space invasion and postoperative CSF leakage (OR = 4.879, 95% CI: 1.243–12.820, *P* = 0.007), and revealed a significantly increased risk of postoperative CSF leakage in patients with incomplete arachnoid structures. In our data, only 4 out of 14 cases of CSF leakage did not develop subarachnoid invasion. Suprasellar tumors, especially tuberculum sellae meningiomas, were difficult to keep the arachnoid intact after lesion removal because of the tumor consistency, and even accompanied with injury to the brain tissue and perforating vessels (19, 20), the risk of CSF leakage is relatively high in these cases. Therefore, we point out that the integrity of the arachnoid is a more influencing factor than the tumor size or suprasellar extension in postoperative CSF leakage, which is consistent with findings of Campero et al. described previously (21).

#### Intraoperative grade 3 flow CSF leakage

Several studies reported that intraoperative CSF leakage was an independent factor of postoperative CSF leakage (11, 22). However, a few literature studies analyzed the postoperative CSF leakage by classifying intraoperative CSF leakage flow. Here, we showed that the incidence of postoperative CSF leakage in patients with intraoperative CSF leakage was 7.1%, which is in accord with the range of 6%–53.2% reported in literature studies (10, 14, 22). The risk of postoperative CSF leakage was significantly higher than that of patients without intraoperative CSF leakage (7.1% vs. 0%). The result suggests that patients with intraoperative CSF leakage are more needed aggressive treatment to prevent postoperative CSF leakage. In addition, some cases in which sellar diaphragm remains intact after tumor removal still developed postoperative CSF leakage, which might be attributed to inadequate postoperative skull base reconstruction or low-flow CSF leakage omitted intraoperatively (23).

Further study of intraoperative flow CSF leakage showed that not all intraoperative CSF leakages were associated with postoperative CSF leakage. Intraoperative grade 1 or grade 2 flow CSF leakage was not statistically correlated with CSF leakage after the operation. It might be related to the fact that the tumor did not invade the suprasellar region, the sellar diaphragm was intact intraoperatively, and CSF was compressed less on the reconstructed structures.

Grade 3 flow CSF leakage was the risk factor (*P* < 0.05) possibly due to a larger defect in the sellar diaphragm in patients with intraoperative grade 3 flow CSF leakage and the intraoperative opening of the suprasellar cistern. Therefore, once extensive leakage was determined, a more aggressive treatment is required to prevent postoperative CSF leakage.

#### Bony reconstruction

*In situ* bone flap or nasal septum bone flap + VP-NSF were used to repair the bony structure of the skull base after September 2018 (Table 5). Among the 104 patients who used bone reconstruction combined with membranous reconstruction, 1 case developed postoperative CSF leakage (0.9%, 1/104) and 4 cases developed intracranial infection (3.8%, 4/104), consistent with previous studies (12), suggesting the reconstruction effect is reliable. Furthermore, the univariate and multivariate analyses results confirmed that bone flap reconstruction was the protective factor of postoperative CSF leakage (OR = 0.313, 95% CI: 0.099–0.694, *P* = 0.019). For patients without CSF leakage during operation, whether to use sellar bone defect reconstruction still remained uncertain. The need for bony reconstruction in patients without intraoperative CSF leakage was still inconclusive, and many surgeons did not consider bony reconstruction as a necessary step when without intraoperative CSF leakage (24). However, for patients with intraoperative CSF leakage, we recommend the use of a bone flap combined with VP-NSF for skull base reconstruction for the following reasons: first, for reconstruction of the outer mucosal layer, the bone flap can theoretically provide mechanical support against the pressure of CSF on the reconstructed site and maintain the original structure of the skull base (13). In addition, bony reconstruction avoids the need for routine postoperative placement of LD, reducing the incidence of retrograde infection, facilitating early postoperative activity, and decreasing the occurrence of venous thrombosis. As to the comparison between *in situ* bone flap and nasal septal bone flap reconstruction in terms of the difference in reconstructive efficacy, there is still no relevant literature report.

**Table 5:** Frequency of postoperative CSF leakage among tumor pathologies.

Tumor pathology	Cases (proportion %)
Pituitary adenoma	8/264 (3.0%)
Craniopharyngioma	4/57 (7.0%)
Meningioma	2/32 (6.3%)

CSF, cerebrospinal fluid.

#### Pathology, tumor size, and other factors

Univariate analysis shows that craniopharyngioma, pituitary adenoma, and meningioma were associated with postoperative CSF leakage. However, this difference was not significant. Pathology did not appear to be correlated with postoperative CSF leakage after multivariate analysis. Furthermore, due to the small sample size, we were unable to compare whether there was a difference in postoperative CSF leakage between pathological types by R × C chi-square test. However, the frequency of postoperative CSF leakage is lower in pituitary adenoma than in craniopharyngiomas and meningioma according to data (Table 5). In addition, larger tumors would theoretically increase the likelihood of invasion of the sellar diaphragm, increasing the postoperative CSF leak rate (7, 25). However, except that the maximum diameter of tumor growth can be located in any axis, tumor does not always invade the arachnoid. Therefore, there was no significant correlation between tumor size and postoperative CSF leakage.

As for other factors including sex, age, diabetes, and hypertension, none of the above was significantly associated with postoperative CSF leakage on adjusted analysis (*P* > 0.05).

### Changes of reconstruction strategy

#### Nasal packing

The main nasal packing support used before was a foley balloon catheter. The multivariate statistical results proved that its usage did not significantly reduce the occurrence of postoperative CSF leakage. Balloon support can significantly reduce the incidence of postoperative CSF leakage for patients with grade 3 flow CSF leakage during operation (26). However, Raza and Schwartz (27) did not suggest using the balloon since it might increase the risk of flap ischemia and cause patient discomfort. However, the author still insists on improving the way of external support. The main reasons are as follows: first, VP-NSF displacement might happen when the foley balloon is placed or extracted. Second, iodoform gauze has uniform pressure distribution and a longer retention time than balloon (14D vs. 7D), which can avoid pulling out the external support when the reconstructed tissue is not completely fibrotic. Finally, it has a certain analgesic effect.

#### Postoperative lumbar drainage

Postoperative LD was used to prevent postoperative CSF leakage based on our experience that a multilayered skull base reconstruction approach with VP-NSF and fascia lata repair alone is inadequate for patients of intraoperative grade 3 flow CSF leakage. However, the statistical results showed that LD was not associated with postoperative CSF leakage. The literature remains unclear on the benefits and risk of postoperative LD as an adjunct in repairing grade 3 flow CSF leakage. Hu et al. (28) advocated routine LD after the operation. Conger et al (29) suggest that LD will cause retrograde infection, low intracranial pressure, and tension pneumocephalus. Others suggest that postoperative LD should be used selectively depending on the location of the skull base defect and the risk factors of CSF leakage (30, 31). In the meantime, a recent meta-analysis revealed that the overall incidence of postoperative CSF leakage in patients who received LD was 7.5%, and the overall incidence of postoperative CSF leakage in patients who did not receive LD was 3.4% (32). All these results suggest that postoperative LD does not reduce the incidence of postoperative CSF leakage. Our data suggest that the bone flap combined with the mucosal flap is sufficient to resist intraoperative grade 3 flow CSF leakage without the need for postoperative LD (12). However, we do not deny the role of postoperative LD in reconstruction strategy. If there is CSF leakage during the repair, we will place LD according to the grade of CSF leakage postoperatively. Thus, we recommend using bone flap combined with VP-NSF for skull base reconstruction in high-risk patients, avoiding routine postoperative using LD.

## Limitations of this study

This study still has some limitations. Preoperative BMI values (33), postoperative intracranial pneumatosis (34), hydrocephalus (35), and intracranial hypertension (36) might be postoperative CSF leakage risk factors. Lucke-Wold et al. (37) suggest that the CSF leakage was associated with multiorganism meningitis. These possible influencing factors were not included in this study. Therefore, the potential factors relate to CSF leakage still need to be studied.

## Conclusion

To summarize, tumor invasion of the subarachnoid space, suprasellar extension, intraoperative grade 3 flow CSF leakage risk factors for postoperative CSF leakage, and bony reconstruction was a protective factor for postoperative CSF leakage. Attention should be paid to patients with high-risk factors. Meanwhile, skull base reconstruction should be given great importance after tumor resection. We recommend using bone flap combined with VP-NSF and iodoform gauze for skull base reconstruction in high-risk patients, avoiding routine postoperative using LD. Patients with suspected postoperative CSF leakage should be explored and repaired promptly.

## Data Availability

The raw data supporting the conclusions of this article will be made available by the authors, without undue reservation.
